# Rapid response team: the early identification of septic patients

**DOI:** 10.1186/cc12933

**Published:** 2013-11-05

**Authors:** Andreia Pardini, Michele Jaures, Sandra Christina Pereira Lima Shiramizo

**Affiliations:** 1 Hospital Israelita Albert Einstein, São Paulo, Brazil

## Background

Rapid response teams (RRTs) represent an intuitively simple concept: when a patient demonstrates signs of imminent clinical deterioration, a team of providers is summoned to the bedside to immediately assess and treat the patient with the goal of preventing ICU transfer, cardiac arrest, or death [1]. Patients whose condition deteriorates acutely while hospitalized often exhibit warning signs (such as abnormal vital signs) in the hours before experiencing adverse clinical outcomes. Sepsis is an illness in which the body has a severe response to bacteria or other germs. This response may be called systemic inflammatory response syndrome (SIRS) [2]. The criteria for calling the RRT are the same as/similar to symptoms of sepsis. We aimed to describe the various criteria for calling the RRT for patients who developed sepsis, initial treatment before transfer to the ICU or step-down unit and outcomes.

## Materials and methods

This retrospective study was conducted in 2012 in the ICU of Hospital Israelita Albert Einstein, a general, private tertiary hospital. During the study period, the hospital had 614 beds, 6.7% (41/614) of which were in the ICU and 13.5% (83/614) were the step-down unit. We included patients 18 years of age or older diagnosed with severe sepsis and septic shock treated by the RRT and transferred to the ICU or step-down unit for study retrospectively. We excluded patients who had contraindications to cardiac resuscitation. Severe sepsis and septic shock were defined according to the American College of Chest Physicians/Society of Critical Care Medicine Consensus Conference definitions [3]. Data regarding age, gender, Simplified Acute Physiology Score II (SAPS II) [4], presence of the following comorbidities, criteria for calling the RRT, initial treatment for sepsis, length of ICU and total stay, and patient outcome were recorded.

## Results

Sixty-five of 41 (63.1%) were males, 23 (35.4%) were transferred to the step-down unit and 42 (64.6%) were transferred to ICU. Their age was 64.7 ± 17.8 years. SAPS II score was 57.8 ± 12.8, length of stay was median 26 days, ICU stay was median 3 days. The treatment of sepsis was also initiated in the ward. The serum lactate + measured blood culture was 40 (63.5%) and fluid administration was 41 (64.1%) (Table 1). Pressing the RRT was in 43 (66.2%) cases by the staff member with significant concern about the patient's condition, 27 (41.5%) cases by changes in systolic blood pressure, and 23 (35.4%) cases due to change in oxygen saturation (Table 2).

**Table 1:** 

Characteristic and outcomes	*n *(%)
Age (years)	64.7 (± 17.8)
Gender (male)	41 (63.1)
Locale of transfer	
ICU	42 (64.6)
Step-down unit	23 (35.4)
Comorbidities	
Cancer	30 (46.2)
Cirrhosis	3 (4.6)
Diabetes	18 (27.7)
Chronic renal failure	11 (16.9)
Chronic obstructive pulmonary disease	6 (9.2)
Chronic heart failure	5 (7.7)
Severe sepsis	56 (86.2)
Septic shock	9 (13.8)
Site of infection	
Pulmonary	29 (44.2)
Urinary tract	13 (20.0
Abdominal	8 (12.3)
Skin and soft tissue	4 (6.2)
Blood (ICS)	4 (6.2)
Others sites	7 (10.8)
SAPS II score	57.8 (± 12.8)
Initial bundle 6 hours	
Serum lactate + blood culture measured	40 (63.5)
Fluids administration	41 (64.1)
Length of stay ICU (days)	3 (1-6)
Length of stay hospital (days)	26 (10-71)
Hospital mortality	13 (20.0)

Data presented as *n *(%), mean (± SD) or median (IQR).

**Table 2:** 

Rapid response system calling criteria	*n *(%)
Heart rate	13 (20.0)
Respiratory rate	8 (12.3)
Systolic blood pressure	27 (41.5)
Oxygen saturation	23 (35.4)
Acute change in mental status	11 (16.9)
Staff member has significant concern about the patient's condition	43 (66.2)

## Conclusions

The criteria for calling the RRT can support the prompt identification of patients who have sepsis and prevent disease progression. Furthermore, the treatment may also be performed in the ward and may result in a reduction in mortality.

## References

[B1] Berwick DM Calkins DR McCannon CJ Hackbarth AD The 100,000 lives campaign: setting a goal and a deadline for improving health care quality JAMA 2006 17 324 327 10.1001/jama.295.3.324 16418469

[B2] Bone RC Balk RA Cerra FB Dellinger RP Fein AM Knaus WA Definitions for sepsis and organ failure and guidelines for the use of innovative therapies in sepsis. The ACCP/SCCM Consensus Conference Committee. American College of Chest Physicians/Society of Critical Care Medicine Chest 1992 17 1644 1655 10.1378/chest.101.6.1644 1303622

[B3] Levy MM Fink MP Marshall JC Abraham E Angus D Cook D 2001 SCCM/ESICM/ACCP/ATS/SIS International Sepsis Definitions Conference Crit Care Med 2003 17 1250 1256 10.1097/01.CCM.0000050454.01978.3B 12682500

[B4] Le Gall JR Lemeshow S Saulnier F A new Simplified Acute Physiology Score (SAPS II) based on a European/North American multicenter study JAMA 1993 17 2957 2963 Erratum: JAMA 1994, **271:**1321 10.1001/jama.1993.03510240069035 8254858

